# Machine learning-based prognostic modeling in gallbladder cancer using clinical data and pre-treatment [^18^F]-FDG-PET-radiomic features

**DOI:** 10.1007/s11604-024-01722-0

**Published:** 2024-12-28

**Authors:** Masatoyo Nakajo, Daisuke Hirahara, Megumi Jinguji, Tetsuya Idichi, Mitsuho Hirahara, Atsushi Tani, Koji Takumi, Kiyohisa Kamimura, Takao Ohtsuka, Takashi Yoshiura

**Affiliations:** 1 Department of Radiology, Graduate School of Medical and Dental Sciences, Kagoshima University, 8-35-1 Sakuragaoka, Kagoshima, 890-8544 Japan; 2 Department of Management Planning Division, Harada Academy, 2-54-4 Higashitaniyama, Kagoshima, 890-0113 Japan; 3 Department of Radiology, Nanpuh Hospital, 14-3 Nagata, Kagoshima, 892-8512 Japan; 4 Department of Digestive Surgery, Graduate School of Medical and Dental Sciences, 8-35-1 Sakuragaoka, Kagoshima, 890-8544 Japan; 5 Department of Advanced Radiological Imaging, Graduate School of Medical and Dental Sciences, Kagoshima University, 8-35-1 Sakuragaoka, Kagoshima, 890-8544 Japan

**Keywords:** Gallbladder cancer, [^18^F]-FDG, Positron emission tomography–computed tomography, Machine learning

## Abstract

**Objectives:**

This study evaluates the effectiveness of machine learning (ML) models that incorporate clinical and 2-deoxy-2-[^18^F]fluoro-D-glucose ([^18^F]-FDG)-positron emission tomography (PET)-radiomic features for predicting outcomes in gallbladder cancer patients.

**Materials and methods:**

The study analyzed 52 gallbladder cancer patients who underwent pre-treatment [^18^F]-FDG-PET/CT scans between January 2011 and December 2021. Twenty-seven patients were assigned to the training cohort between January 2011 and January 2018, and the data randomly split into training (70%) and validation (30%) sets. The independent test cohort consisted of 25 patients between February 2018 and December 2021. Eight clinical features (T stage, N stage, M stage, Union for International Cancer Control [UICC] stage, histology, tumor size, carcinoembryonic antigen level, and carbohydrate antigen 19-9 level) and 49 radiomic features were used to forecast progression-free survival (PFS). Three feature selection methods were applied including the univariate statistical feature selection test method, least absolute shrinkage and selection operator Cox regression method and recursive feature elimination method, and two ML algorithms (Cox proportional hazard and random survival forest [RSF]) were employed. Predictive performance was assessed using the concordance index (C-index).

**Results:**

Two clinical variables (UICC stage, N stage) and three radiomic features (total lesion glycolysis, grey-level size-zone matrix_grey level non-uniformity and grey-level run-length matrix_run-length non-uniformity) were identified by the statistical feature selection method as significant for PFS prediction. The RSF model incorporating these features demonstrated strong predictive performance, with C-indices above 0.80 in both training and testing sets (training 0.81, testing 0.89). This model almost closely matched the actual and predicted progression timelines with a low mean absolute error of 1.435, a median absolute error of 0.082, and a root mean square error of 2.359.

**Conclusion:**

This study highlights the potential of using ML approaches with clinical and pre-treatment [^18^F]-FDG-PET radiomic data for predicting the prognosis of gallbladder cancer.

**Supplementary Information:**

The online version contains supplementary material available at 10.1007/s11604-024-01722-0.

## Introduction

Gallbladder cancer, the leading cancer of the biliary tract, constitutes 1.2% of all cancer cases worldwide [1]. This malignancy is highly aggressive, resulting in poor prognostic outcomes, with an overall 5-year survival rate near 10% [2, 3]. Surgery remains the only curative option. In its advanced stages, the cancer is characterized by local tissue invasion, widespread regional lymph node metastasis, and distant metastasis, contributing to poor outcomes due to non-resectable or metastatic disease [4, 5].

Cross-sectional imaging like computed tomography (CT) scan and magnetic resonance imaging provides detailed anatomical information crucial for staging and treatment planning in gallbladder cancer [6, 7]. However, relying solely on these images may not be sufficient to predict treatment outcomes and prognosis. Additional reliable data, such as biological or molecular markers, might be required for better prognostic prediction and treatment strategy formulation in gallbladder cancer.

Radiomics utilizes mathematical approaches to derive quantitative features that provide valuable biological data [8–10]. In oncology, positron emission tomography (PET)/CT scanning with the glucose analog 2-deoxy-2-[^18^F]fluoro-D-glucose ([^18^F]-FDG) is a common method for assessing metabolic activity [11]. Studies have shown the effectiveness of [^18^F]-FDG-PET/CT scans in the diagnosis, staging, and prediction of treatment response and prognosis in gallbladder cancer [12–14]. To date, the prognostic value of [^18^F]-FDG-PET-based radiomic data in gallbladder cancer has not been thoroughly investigated.

Traditional statistical approaches rely on predefined models, while machine learning (ML) emphasizes discovering intricate patterns among variables through data-driven learning with evolving algorithms to enhance prediction accuracy [15, 16]. Recent researches have applied ML and deep learning in nuclear medicine [10, 17–21]. In addition, artificial intelligence has been employed to enhance image quality in this field, and large language models are expected to contribute to the standardization of medical reports and electronic health records [10]. However, the effectiveness of [^18^F]-FDG-PET-based radiomics for predicting outcomes in gallbladder cancer using ML has not been studied.

Our research aimed to explore the prognostic utility of ML approaches, incorporating clinical and [^18^F]-FDG-PET-based radiomic data, in patients with gallbladder cancer.

## Materials and methods

### Patients

This single-center retrospective study received approval from our institutional review board, with a waiver for written informed consent. A total of 104 patients, either suspected or confirmed to have primary gallbladder cancer, underwent pre-treatment [^18^F]-FDG-PET/CT scans between January 2011 and December 2021. Clinical records were subsequently reviewed to select eligible patients for analysis.

Eligibility criteria included: (1) patients with histologically confirmed gallbladder cancer; (2) absence of prior radiotherapy, chemoradiotherapy, or chemotherapy before surgery; and (3) a primary tumor with detectable uptake on PET/CT imaging. Exclusion criteria were: (1) presence of other concurrent malignancies; (2) a small primary tumor making texture analysis difficult (volume of interest [VOI] <64 voxels; Discovery 600M scanner: less than 2.04 ml or Discovery MI scanner: less than 1.20 ml); and (3) incomplete follow-up records.

**Fig. 1:**
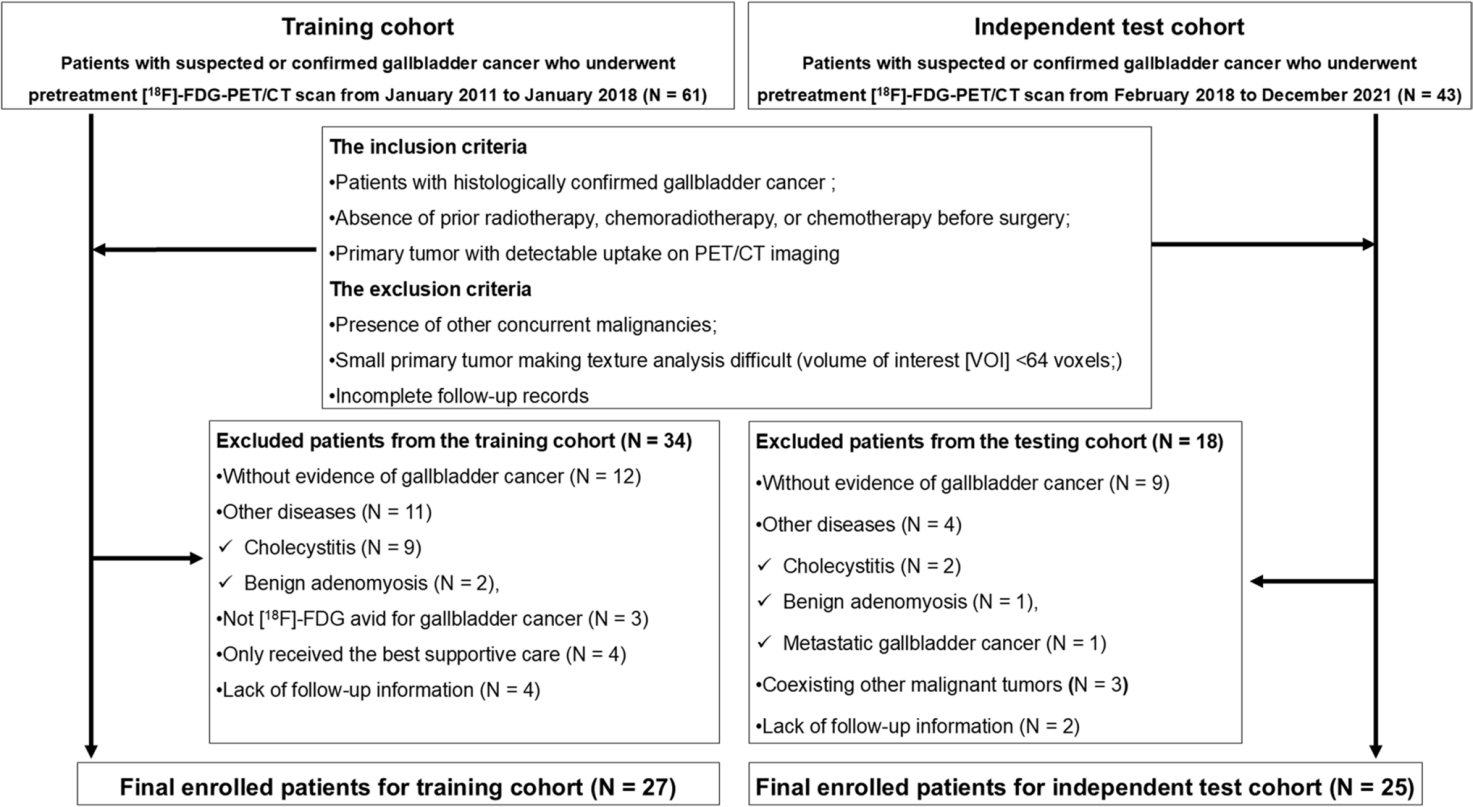
Flowchart of the study patient selection steps

The flowchart in Fig. 1 illustrates the patient selection process for the study. The training cohorts consisted of 61 patients suspected of or diagnosed with primary gallbladder cancer who underwent a pre-treatment [^18^F]-FDG-PET/CT scan between January 2011 and January 2018. Among them, 12 without gallbladder cancer and physiological [^18^F]-FDG uptake and 11 with other conditions (9 cholecystitis, 2 adenomyosis) were excluded. In addition, three patients with non-avid [^18^F]-FDG lesions, four receiving best supportive care, and four lacking follow-up data were excluded. Finally, 27 patients (12 men and 15 women; mean age: 69 ± 10 years, range: 51–92 years) met the criteria for the training cohorts, and the development of the ML models was performed on this training cohort.

The testing cohort was reserved to perform the external test for estimating the final predictive performances.

The independent external test cohort consisted of 43 patients suspected of or diagnosed with primary gallbladder cancer who underwent a pre-treatment [^18^F]-FDG-PET/CT scan between February 2018 and December 2021. Among them, nine without gallbladder cancer and physiological [^18^F]-FDG uptake and four with other conditions (2 cholecystitis, 1 adenomyosis, 1 metastatic gallbladder cancer) were excluded. In addition, three with coexisting malignancies (2 rectal cancers, 1 sarcoma) and two with lacking follow-up data were excluded. Finally, 25 patients (12 men and 13 women; mean age: 72 ± 9 years, range: 56–90 years) were assigned as the independent external test cohort.

### Imaging protocols

The PET/CT scans were conducted using two different whole-body PET/CT systems. From January 2011 to January 2018, the Discovery 600M PET/CT scanner (GE Healthcare, Milwaukee, WI, USA) was utilized, and from February 2018 to December 2021, the Discovery MI scanner (GE Healthcare) was employed. Patients fasted for at least 5 h before the scan (mean plasma glucose level ± SD: 110 ± 22 mg/dL, range: 83–169 mg/dL). Intravenous administration of [^18^F]-FDG (FDG Scan; Nihon Medi-Physics, Tokyo, Japan) was performed. A PET/CT emission scan was conducted 1-h post-injection of [^18^F]-FDG (mean ± SD: 222 ± 33 MBq, range: 159–276 MBq), following the CT data acquisition (parameters: 3.75 mm slice thickness, 1.375 mm pitch, 120 keV, and auto mA adjustment between 40 and 100 mA based on body mass). The acquisition time was 2.5 min per bed position, totaling 7–11 positions. Attenuation-corrected data were collected. Images from the Discovery 600M scanner were reconstructed using a 3D ordered subset expectation-maximization algorithm, with an image matrix of 192 × 192, 16 subsets, and two iterations, producing voxel sizes of 3.125 × 3.125 × 3.27 mm^3^ using VUE Point Plus. For the Discovery MI scanner, images were reconstructed using time of flight (TOF) with a Bayesian penalized likelihood algorithm (Q.Clear), featuring a matrix size of 192 × 192, voxel sizes of 2.60 × 2.60 × 2.78 mm^3^, and a penalization factor of 700, incorporating point spread function modeling. Consistent reconstruction settings and matrix were applied for each scanner.

### Image and radiomic feature analyses

Two radiologists, with 12 and 19 years of experience in [^18^F]-FDG-PET/CT scans, respectively, were informed of the study’s purpose but were blinded to clinical and pathological details. They reached a consensus on whether the primary lesion exhibited abnormal [^18^F]-FDG uptake, defined as uptake exceeding the background activity of surrounding tissues. A third radiologist, with 17 years of experience in [^18^F]-FDG-PET/CT, conducted quantitative analysis on the primary visible lesions. This radiologist manually set the volume of interest (VOI) on a reference-fused axial image, determining the craniocaudal and mediolateral boundaries to include the entire visible lesion while excluding any nearby physiological [^18^F]-FDG-avid tissues. The VOI boundaries were set using a threshold of 40% of the maximum standardized uptake value (SUVmax). The LIFEx software (version 7.2) [22] was employed to derive 49 radiomic features from the PET images (Supplemental Table 1). This software requires VOIs to contain a minimum of 64 voxels to compute textural features. To mitigate correlations between features and reduce noise effects as well as matrix size, both the VOI and SUV values were resampled into discrete bins using an absolute resampling approach [23]. For the PET component resampling, a total of 64 bins were created, covering an SUV interval from 0 to 20. Voxel dimensions were standardized to 3.0 × 3.0 × 3.0 mm^3^, resulting in a bin size of 0.3 SUV. Any voxel with an SUV higher than 20 was assigned to the uppermost bin [23]. Since two PET scanners were utilized, we harmonized the PET parameters post-reconstruction using the ComBat method within R software (https://github.com/Jfortin1/ComBatHarmonization) [24], which has been validated in previous PET research [25].

### Treatment and follow-up

For each patient, staging of the tumor was conducted using the TNM classification system (7^th^ edition) by the UICC, based on standard pre-treatment clinical examinations and imaging studies, including [^18^F]-FDG-PET/CT. The results from these assessments guided the treatment strategy (Supplemental Material).

The prognosis of each patient was determined using medical records, with the last follow-up recorded in December 2023. Progression-free survival (PFS) was measured from the start of treatment to disease progression, death from cancer, or the date of the last follow-up, whichever was earlier.

### ML approach

**Fig. 2:**
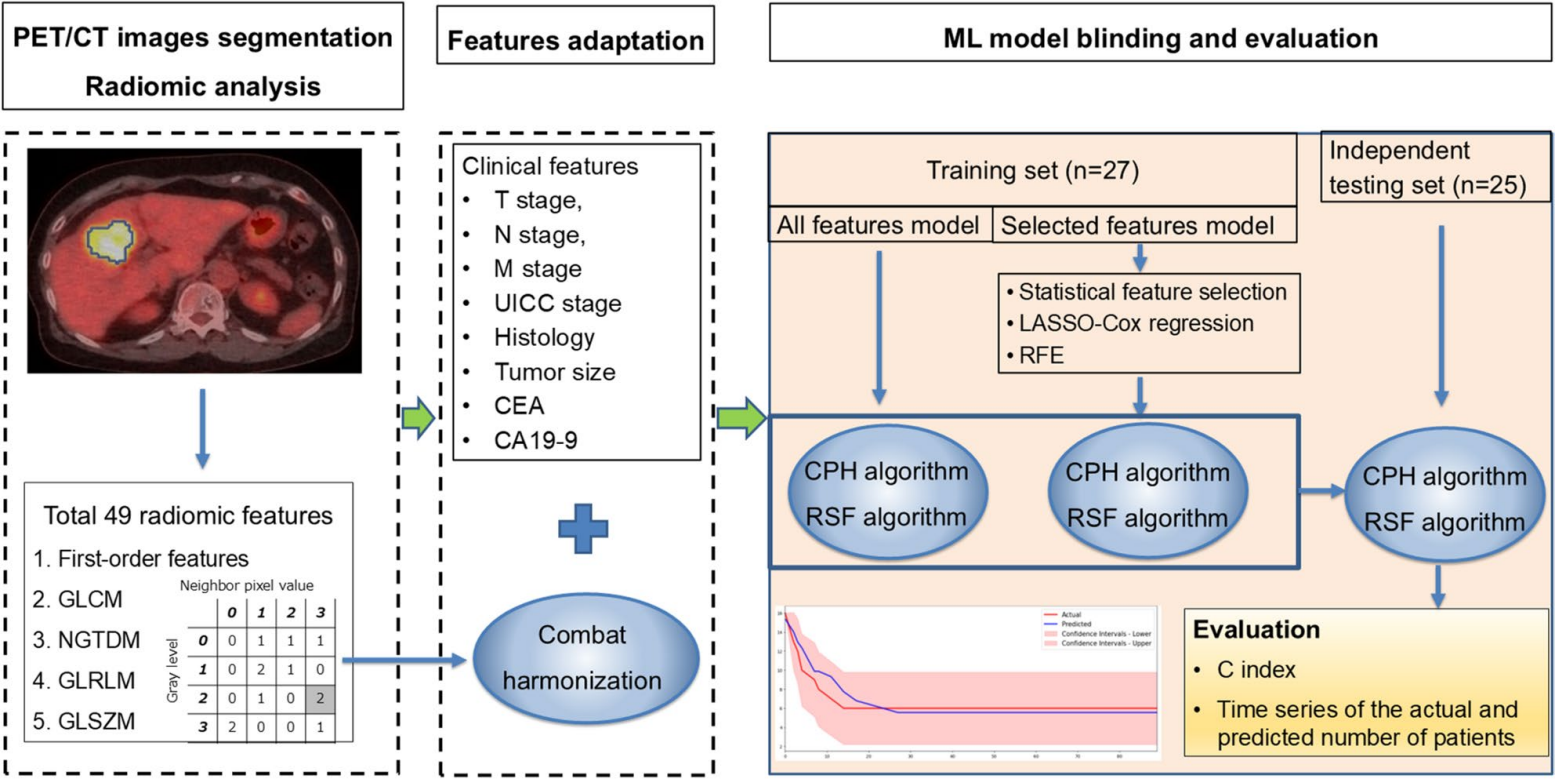
Work flow of the present study. Radiomics features have been extracted from the volume of interest segmented from PET/CT images. Then ComBat harmonization is applied to each radiomic feature due to two different PET scanners. Finally, two ML models (all features ML model and selected features ML model) are developed to predict PFS of the patients with gallbladder cancer using clinical features and PET/CT radiomic features

The workflow of this study, depicted in Fig. 2, utilized eight clinical factors (T stage, N stage, M stage, UICC stage, histology, tumor size and two biomarkers [carcinoembryonic antigen (CEA) and carbohydrate antigen 19-9 (CA19-9)]) and 49 radiomic features to assess PFS with ML approaches.

In the training cohort, the data were categorized by event and randomly split into training (70%) and validation (30%) sets. ML models were employed to analyze time-to-event data, specifically using the linear cox proportional hazard (CPH) model and the nonlinear random survival forest (RSF) approach, with detailed settings available in the Supplemental Material.

With a small sample size, feature reduction was necessary to minimize overfitting. In the training cohort, three feature selection methods were applied: univariate statistical feature selection based on the Mann–Whitney *U* test [26], least absolute shrinkage and selection operator (LASSO) Cox regression [27], and recursive feature elimination (RFE) [28]. The Mann–Whitney *U* test, a non-parametric method, assesses differences between two independent groups to identify features with significantly different distributions [26]. The LASSO method is used in data analysis to minimize the coefficients of variables that are not associated with survival, effectively reducing them to zero [29]. It identifies critical features by suppressing the influence of unimportant ones, removing redundancy, and selecting variables with non-zero coefficients for model development [27]. RFE systematically evaluates and discards features to determine those that best enhance model accuracy [28]. Both full-feature sets and selected sets were used for ML model construction, adhering to the principle of using fewer than 10% of the sample size as features for classification [30]. The study’s total sample size was n=52, leading us to select under five features for the ML model. Feature important score was used to evaluate the effect of the features for the ML model development [31]. In addition, to address potential overfitting, we applied k-fold cross-validation, specifically a fivefold approach, to reduce overfitting effects [32–34].

The predictive power of the ML models was evaluated using the concordance index (C-index), which determines how well the predicted event times correspond with actual patient outcomes. A C-index of 0.5 reflects random chance, while a value of 1 represents perfect accuracy [35, 36]. Typically, a C-index over 0.8 indicates strong predictive performance [35, 36].

The performance of the predictive models was assessed in the testing cohort using root mean squared error and mean/median absolute errors. These metrics compared actual versus predicted progression events [37, 38]. Within the testing cohort, each ML algorithm computed the PFS probability. The median survival time, representing a 50% PFS probability, was utilized to evaluate the precision of survival predictions, where closer proximity to the actual survival time indicated better performance [38]. The PySurvival library was employed for conducting this survival ML analysis [39]. In addition to PySurvival, we used scikit-learn for feature selection and data scaling.

### Statistical analysis

The study utilized the Mann–Whitney *U* test or Chi-square test to analyze differences between variables and categorical data. Kaplan–Meier survival curves were drawn, with log-rank tests used to determine significance. Data were presented as medians with interquartile range (IQR). Statistical significance was established at *p* < 0.05, with two-sided *p* values considered. Analyses were performed using MedCalc software (MedCalc, Mariakerke, Belgium).

## Results

### Characteristics of the patients

Twenty-seven patients assigned to the training cohort underwent PET/CT imaging on the Discovery 600-M scanner, with 25 adenocarcinoma cases and two neuroendocrine carcinoma cases identified. Meanwhile, the testing cohort of 25 patients underwent imaging on the Discovery MI scanner, including 22 adenocarcinoma cases, two adenosquamous carcinoma cases, and one neuroendocrine carcinoma case. Treatment details are included in the Supplemental Material. Table 1 presents the clinical characteristics of the training and testing cohorts. Of the 27 patients in the training cohort, 19 experienced disease progression, resulting in 19 deaths. In the testing cohort, 14 of 25 patients experienced disease progression, with nine deaths and five survivors. There were significant differences (*p* < 0.05) in treatment method, T, N, and UICC stages between the non-progression and progression groups in both cohorts (Table 1).

**Table 1.** Characteristics of patients with gallbladder cancer (*n* = 52)

Characteristics of the patients	Training cohort (*n* = 27)			Testing cohort (*n* = 25)			*p* value^b^
	Discover 600M scanner			Discover MI scanner			
	Non-progression group	Progression group	*p* value^a^	Non-progression group	Progression group	*p* value^a^	
Number	8	19		11	14		
Age (years) (mean, range)	69, 55–85	68, 51–92	0.85	74, 56–90	70, 57–81	0.41	0.29
Sex							
Male	2	10	0.20	5	7	0.82	0.80
Female	6	9		6	7		
Treatment method			0.001			0.003	0.006
Surgery alone	6	1		10	3		
Surgery with adjuvant therapy	2	11		0	2		
Chemotherapy	0	7		1	9		
Tumor size (mm) (median, range)	49.9 (30.4–68.5)	39.5 (22.3–90.0)	0.33	30.9 (25.1–42.6)	51.9 (32.2–106.6)	0.002	0.99
Histology			0.35			0.70	0.58
Adenocarcinoma	8	17		10	12		
Others	0	2		1	2		
Tumor marker							
CEA level (ng/mL) (median, range)	2.85 (1.0–39.3)	2.6 (0.5–46.4)	0.85	1.5 (1.0–9.8)	4.2 (0.8–982.0)	0.010	0.93
CA19-9 level (U/mL) (median, range)	10.8 (2.0–155.2)	12.9 (0.9–420.8)	0.83	8.1 (6.6–40.3)	44.1 (0.6–9675.0)	0.028	0.64
T stage			0.035			<0.001	0.33
1	1	0		5	0		
2	4	5		6	2		
3	3	12		0	8		
4	0	2		0	4		
N stage			<0.001			<0.001	0.28
0	7	3		11	2		
1	1	16		0	12		
M stage			0.31			0.007	0.87
0	7	13		11	7		
1	1	6		0	7		
UICC stage			0.004			<0.001	0.24
I	1	0		5	0		
II	4	0		6	0		
IIIA	2	3		0	1		
IIIB	0	9		0	5		
IVA	0	1		0	1		
IVB	1	6		0	7		

^a^ Comparison of the non-progression and progression groups in the training and testing cohorts

^b^ Comparison of the training and testing cohorts

*CEA* carcinoembryonic antigen, *CA19-9* carbohydrate antigen 19-9

In addition, significant differences were observed between the non-progression and progression groups in the testing cohort concerning tumor size, CEA, CA19-9 level, and M stage (each, *p* < 0.05), while these differences were not significant in the training cohort (each, *p* > 0.05). No significant differences were noted between the groups in either cohort regarding age, sex, or histology (each, *p* > 0.05) (Table 1).

The training and testing cohorts showed no significant variation except for treatment method in terms of age, sex, tumor size, histology, tumor marker concentrations, and TNM stages, including UICC classification.

### ML methods for progression survival analyses

**Fig. 3:**
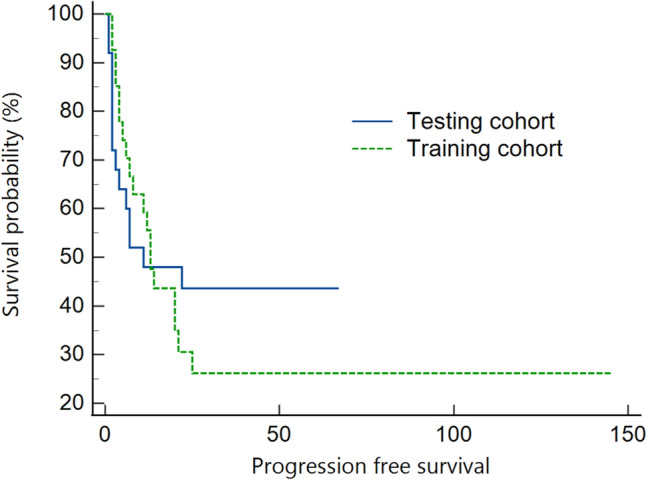
Kaplan–Meier survival curves of the training and testing cohorts. There was no significant difference in terms of PFS between the training and testing cohorts (*p* = 0.62)

The average duration of follow-up was 56.3 months overall (range: 1–145 months), with the training cohort at 45.8 months (range: 2–145 months) and the testing cohort at 32.2 months (range: 1–67 months). Median PFS times were 13.0 months for all patients, 13.0 months for the training cohort, and 11.0 months for the testing cohort. The 5-year PFS rates were 35.1% overall, 26.2% in the training cohort, and 43.6% in the testing cohort, with no significant difference in PFS between cohorts (Fig. 3) (*p* = 0.62).

For the construction of the ML model to predict PFS, the statistical feature selection method identified five key features based on their importance scores: N stage, UICC stage, total lesion glycolysis (TLG), grey-level size-zone matrix (GLSZM)_grey-level non-uniformity (GLNU), and grey-level run-length matrix (GLRLM)_run-length non-uniformity (RLNU) (Supplementary Fig. 1). Using LASSO Cox regression, the selected features included GLSZM_zone percentage (ZP), GLSZM_normalized zone-size non-uniformity, histology, compacity, and N stage (Supplementary Fig. 2). The RFE method selected another set of five features: histology, N stage, UICC stage, compacity, and GLSZM_ZP (Supplementary Fig. 3).

The C-index outcomes for each survival ML model are summarized in Table 2. The CPH algorithm produced C-indices of 1.00 for the full-feature model, 0.71 for the model using statistical feature selection, 0.82 for the model based on LASSO Cox regression, and 0.80 for the RFE-based model in the training cohort. The RSF algorithm demonstrated C-indices of 0.82, 0.81, 0.81, and 0.81 for these models.

**Table 2:** C-index of each machine learning model in the training and testing cohorts for predicting progression-free survival in gallbladder cancer

Algorithm	Full-features model		Selected features model					
			Statistical		LASSO		RFE	
	Training cohort	Testing cohort	Training cohort	Testing cohort	Training cohort	Testing cohort	Training cohort	Testing cohort
Linear Cox proportional hazard model	1.00	0.49	0.71	0.91	0.82	0.81	0.80	0.79
Random survival forest	0.82	0.85	0.81	0.89	0.81	0.79	0.81	0.83

*LASSO* least absolute shrinkage and selection operator, *RFE* recursive feature elimination

In the testing cohort, the CPH algorithm recorded C-indices of 0.49 for the full-feature model, 0.91 for the statistical feature selection method, 0.81 for the LASSO Cox regression method, and 0.79 for the RFE method. Meanwhile, the RSF algorithm produced C-indices of 0.85, 0.89, 0.79, and 0.83 across the same models. Among the ML models with C-indices exceeding 0.80 in both training and testing sets, the selected feature ML model built using the statistical feature selection method with the RSF algorithm achieved the highest C-index of 0.89 in the testing cohort. According to the results of feature importance score, N stage, UICC stage, TLG, GLSZM_GLNU, and GLRLM_RLNU showed higher contribution for developing this best predictive ML model (Supplementary Fig. 1). Notably, patients who experienced disease progression exhibited significantly elevated TLG, GLSZM_GLNU, and GLRLM_RLNU levels compared to those without progression (each, *p* < 0.05) (Supplemental Table 2).

**Fig. 4:**
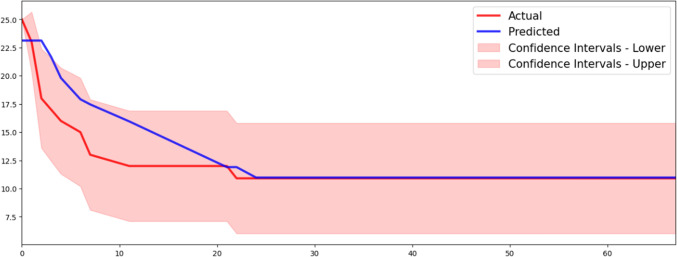
The actual number of patients with disease progression versus the predicted number of patients with disease progression estimated using the selected ML model with RSF algorithm by the statistical feature selection method. The time series of the actual and predicted number of patients with disease progression are nearly similar, with a low mean absolute error of 1.435, median absolute error of 0.082, and root mean square error of 2.359

In the testing cohort, a comparison between the actual and predicted patient counts with disease progression was conducted using the abovementioned best predictive PFS model. The results demonstrated almost nearly close alignment, with a low mean absolute error of 1.435, a median absolute error of 0.082, and a root mean square error of 2.359 (Fig. 4).

**Fig. 5:**
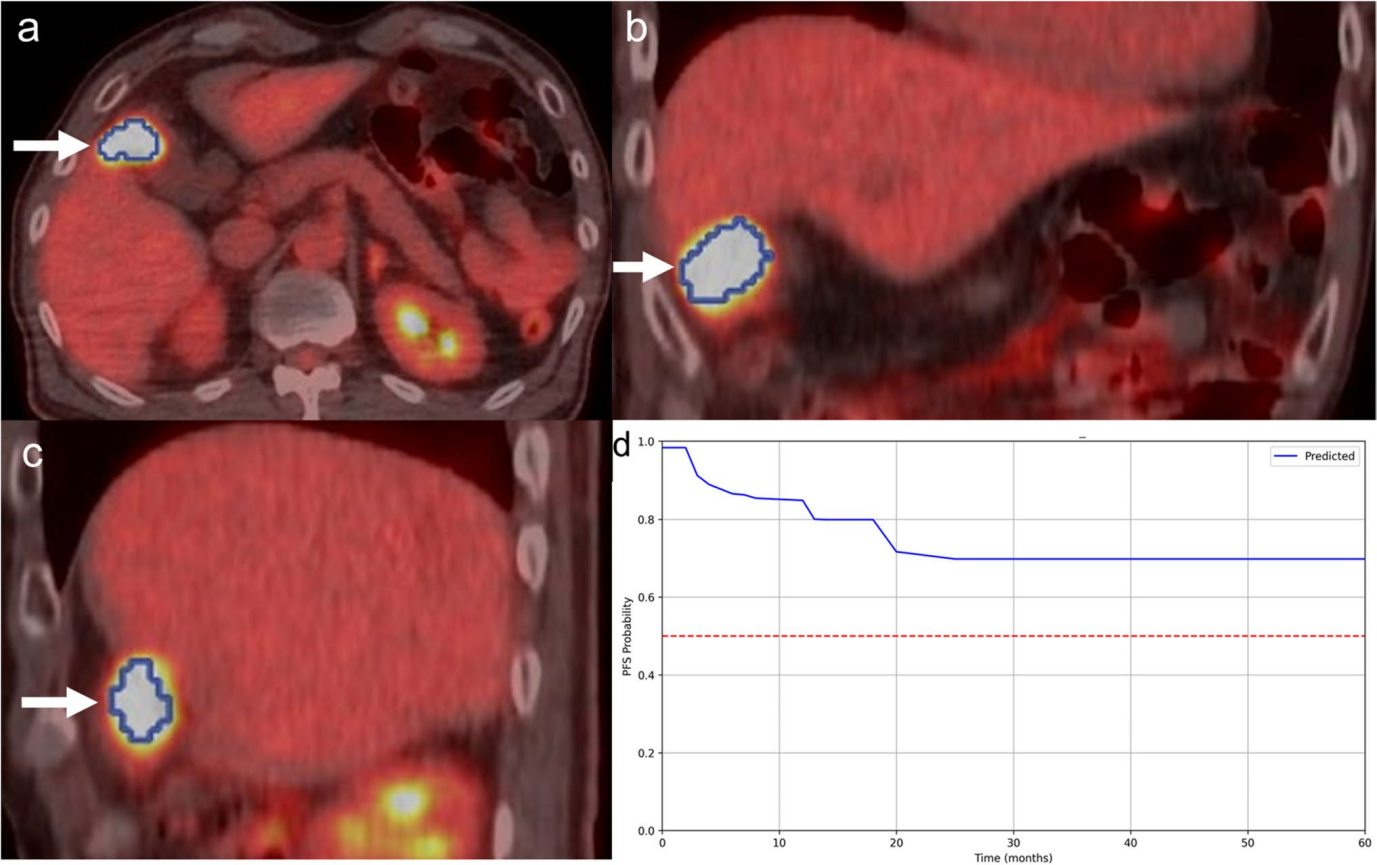
A 67-year-old man with gallbladder cancer (adenocarcinoma, T2N0M0, stage II) had no progression after surgery. Pretreatment [^18^F]-FDG-PET/CT [transaxial (**a**), coronal (**b**), and sagittal (**c**)] images revealed abnormal [^18^F]-FDG uptake in the primary lesion. The blue line represents the border of the VOI (arrows). The personalized survival prediction using the selected features ML model by the statistical feature selection method with the RSF algorithm (the best performing ML algorithm) is presented (**d**). The X-axis represents the survival time in months, and the Y-axis indicates the PFS probability. The PFS probability was 69.8% at 50 months after the surgery. The PFS probability did not reach 50%, and the ML algorithm predicted non-progression at this time. Indeed, the patient survived without progression for 50 months after the surgery

**Fig. 6:**
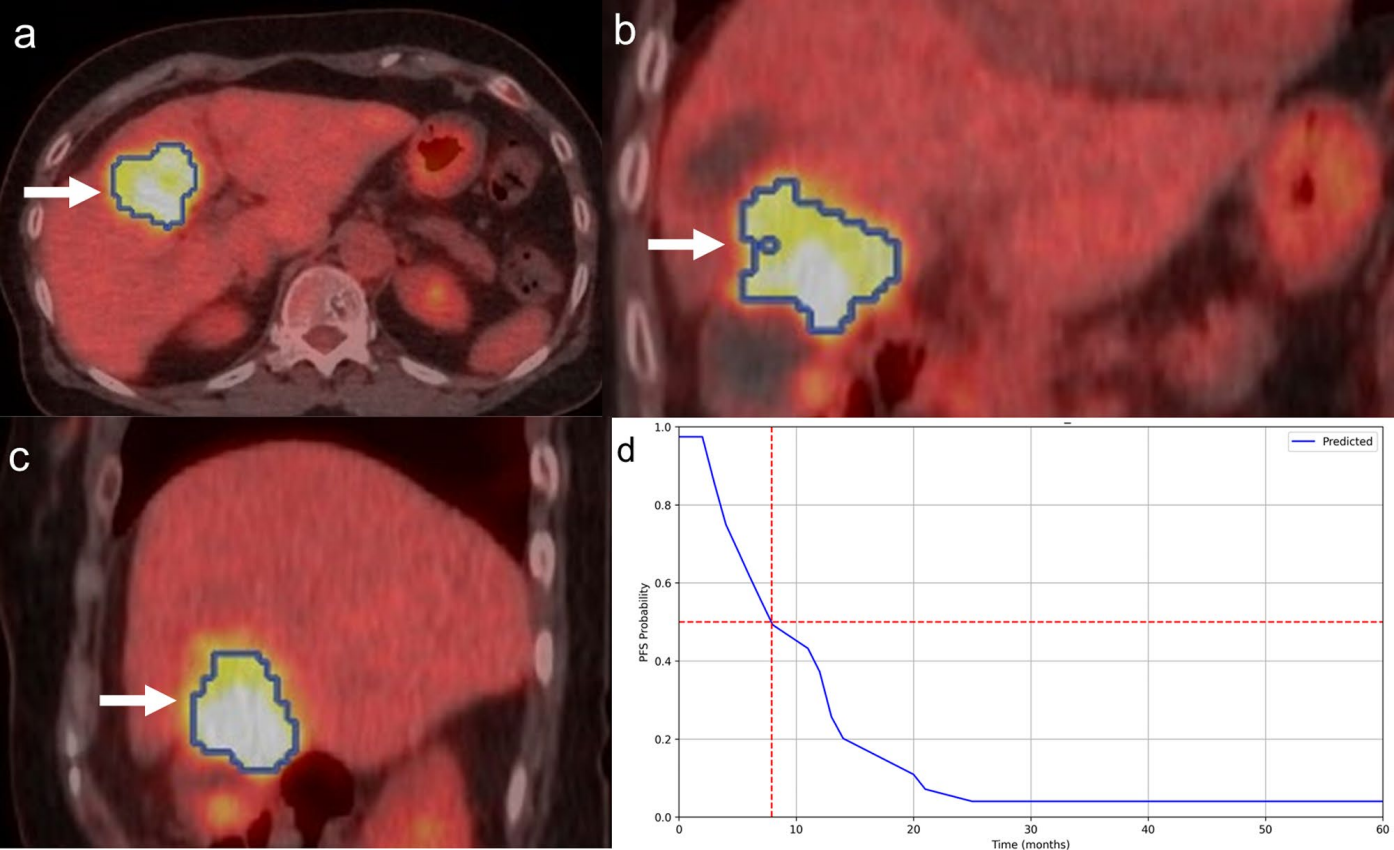
A 71-year-old woman with gallbladder cancer (adenocarcinoma, T3N1M0, stage IIIB) had progression after chemotherapy. Pretreatment [^18^F]-FDG-PET/CT scan [transaxial (**a**), coronal (**b**), and sagittal (**c**)] images revealed an abnormal [^18^F]-FDG uptake in the primary lesion. The blue line represents the border of the VOI (arrows). The personalized survival prediction based on the selected features ML model by the statistical feature selection method with the RSF algorithm (the best performing ML algorithm) is presented (**d**). The X-axis represents survival time in months, and the Y-axis indicates PFS probability. The 50% PFS probability was at 7.8 months (dotted line, **d**). Although the patient received chemotherapy, she developed progressive disease 4 months after therapy initiation. The patient’s 50% PFS probability was almost close to 4 months, and the ML algorithm correctly predicted the progression

Figures 5 and 6 display the [^18^F]-FDG-PET/CT scan images of patients showing no disease progression and those with progression, respectively.

## Discussion

The current study examined the usefulness of the ML approach using pre-treatment clinical and [^18^F]-FDG-PET-based radiomic features for predicting PFS in patients with gallbladder cancer. The RSF-driven statistical feature selection approach produced an ML model with the best predictive performance for PFS. N stage, UICC stage, TLG, GLSZM_GLNU, and GLRLM_RLNU had a higher contribution in this modeling processing. Thus, ML analyses using pre-treatment clinical and [^18^F]-FDG-PET-based radiomic features may be useful for predicting PFS in patients with gallbladder cancer.

Several recent studies have developed ML-based classification methods in nuclear medicine [10, 17–21]. Lue et al. [21] reported the usefulness of deep learning methods using ^18^F-FDG-PET/CT for predicting prognosis in patients with EGFR-mutated lung adenocarcinoma. Toyama et al. [40] found that GLSZM_GLNU was the most significant factor for predicting 1-year survival in pancreatic cancer patients using ML models on [^18^F]-FDG-PET/CT images. To date, no research has explored the use of [^18^F]-FDG-PET radiomics with ML for prognostic prediction in gallbladder cancer. Our analysis included 8 clinical and 49 radiomic features to predict PFS using ML techniques. The ML model developed using the RSF-based statistical feature selection method stood out among models with C-indices above 0.80 in training and testing sets, achieving the highest C-index of 0.89 in the testing cohort. This performance underscores its predictive precision for PFS. By incorporating two clinical variables (N stage and UICC stage) and three radiomic variables (TLG, GLSZM_GLNU, and GLRLM_RLNU), this model closely approximated the actual progression timelines in comparison to the predictions. Thus, ML analysis with these pre-treatment features may be valuable in forecasting PFS in gallbladder cancer patients.

Several studies have explored [^18^F]-FDG radiomic features, including TLG, GLSZM_GLNU, and GLRLM_RLNU, in not only gallbladder cancers but also other malignancies [41–46]. Yoo et al. [42] reported that high TLG were associated with poor overall survival (OS) in patients with gallbladder cancer. GLSZM_GLNU indicates the dispersion of zone counts across grey levels, where a lower value suggests uniformity [43]. GLRLM_RLNU, a feature from the GLRLM family, quantifies the variability in run lengths; when runs have similar lengths, RLNU values are low [44]. Heterogeneous images usually result in higher GLSZM_GLNU values [43] and higher GLRLM_RLNU [44]. Chen et al. [45] explored the role of [^18^F]-FDG-PET radiomics in predicting treatment outcomes for oro- and hypo-pharyngeal cancer. They found that a higher GLSZM_GLNU value was linked to poorer cause-specific survival. According to Ho et al. [46], elevated GLRLM_RLNU values were linked to worse overall survival (OS) in patients with bulky cervical cancer undergoing CRT. Similarly, our study revealed that patients with disease progression exhibited higher TLG, GLSZM_GLNU, and GLSZM_RLNU values, suggesting that increased and heterogeneous [^18^F]-FDG uptake in primary tumors may be associated with poorer outcomes in gallbladder cancer.

This study has certain limitations. First, it is retrospective with a relatively small sample size, which may lead to selection bias. A larger, prospective study is necessary for validation. Second, because our data from a single institution may not be generalizable, the validation with other institutions is warranted in further studies. Moreover, while the selected features of ML model achieved satisfactory results in the training and independent external testing phases, a larger dataset may be needed for comprehensive validation. Third, variability in treatment method could have influenced the survival analysis results. However, the treatment methods were not included for construction ML models to avoid the risks of circular reasoning, and the ML models were constructed by only pre-treatment features. Finally, two different PET/CT scanners were used. Disparities in performance levels, such as imaging techniques like TOF, were observed between the two PET/CT scanners. Furthermore, the threshold for segmented VOI volume varied between them. Therefore, the lack of image-based harmonization could have impacted the results of [^18^F]-FDG-PET-based radiomic analyses. While image-based harmonization is crucial, it necessitates post-processing that can diminish spatial resolution in images from advanced devices, leading to less-than-ideal image quality for subsequent quantitative and radiomic analyses [25]. The implementation of the ComBat algorithm for post-reconstruction harmonization in [^18^F]-FDG-PET-based radiomic studies has proven beneficial [25, 47]. According to Orlhac et al. [25], ComBat effectively addressed batch effects arising from vendor and imaging protocol differences in [^18^F]-FDG-PET/CT images for both triple-negative and non-triple-negative breast cancers. Furthermore, Dissaux et al. [47] demonstrated similar benefits in non-small cell lung cancer survival prediction using different [^18^F]-FDG-PET/CT images. In our analysis, significant differences in SUVmax and SUVmean distributions for the liver, representing a normal organ, were observed between the two PET/CT scanners prior to ComBat harmonization (Supplemental materials, Supplemental Table 3, and Supplemental Figs. 4 and 5). Following harmonization, these discrepancies were resolved, leading to improved overlap in the distributions (Supplemental Table 3 and Supplemental Figs. 4 and 5). As there were no significant differences in clinical parameters between the scanners (Table 1), ComBat harmonization could be performed without incorporating biological covariates.

In conclusion, this study highlights the potential of using ML approaches with clinical and pre-treatment [^18^F]-FDG-PET radiomic data for predicting the prognosis of gallbladder cancer.

## Supplementary Information

Below is the link to the electronic supplementary material.

Supplementary file1 (DOCX 628 KB)
