# Supplementary material for: Machine learning-based prognostic modeling in gallbladder cancer using clinical data and pre-treatment [18F]-FDG-PET-radiomic features
Source: Jpn J Radiol. 2024 Dec 28;43(5):864–74. doi: 10.1007/s11604-024-01722-0 (PMC12053127; doi:10.1007/s11604-024-01722-0)
Supplement: Supplementary file 1 — Supplementary file1 (DOCX 628 KB) [file 11604_2024_1722_MOESM1_ESM.docx]

| **Supplemental Table 1.** Complete list of 49 quantitative positron emission tomography-based radiomic features | |
| --- | --- |
| Matrix | Index |
| Shape and first order features | SUV_max_ |
|  | SUV_mean_ |
|  | SUV_peak_ |
|  | Metabolic tumor volume |
|  | Total lesion glycolysis |
|  | Surface |
|  | Sphericity |
|  | Asphericity |
|  | Compacity |
|  | Kurtosis |
|  | Skewness |
| Grey-level co-occurrence matrix | Inverse difference |
|  | Angular second moment |
|  | Contrast |
|  | Correlation |
|  | Entropy |
|  | Dissimilarity |
| Neighborhood grey-level different matrix | Coarseness |
|  | Contrast |
|  | Busyness |
|  | Complexity |
|  | Strength |
| Grey-level run-length matrix | Sort-runs emphasis |
|  | Long-runs emphasis |
|  | Low-gray-level run emphasis |
|  | High-gray-level run emphasis |
|  | Short-run low-gray level emphasis |
|  | Short-run high-gray level emphasis |
|  | Long-run low-gray level emphasis |
|  | Long-run high-gray level emphasis |
|  | Gray-level non-uniformity for run |
|  | Run-length non-uniformity |
|  | Run percentage |
| Grey-level size-zone matrix | Short-zone emphasis |
|  | Long-zone emphasis |
|  | Low-gray-level zone emphasis |
|  | High-gray-level zone emphasis |
|  | Small-zone low-gray level emphasis |
|  | Small-zone high-gray level emphasis |
|  | Large-zone low-gray level emphasis |
|  | Large-zone high-gray level emphasis |
|  | Gray-level non-uniformity |
|  | Normalized gray-level non-uniformity |
|  | Zone-size non-uniformity |
|  | Normalized zone-size non-uniformity |
|  | Zone percentage |
|  | Gray-level variance |
|  | Zone-size variance |
|  | Zone-size entropy |

| **Supplemental Table 2.** Comparison of important PET radiomic features between the non-progression and progression groups | | | | | | | |
| --- | --- | --- | --- | --- | --- | --- | --- |
|  | Non-progression group (n = 19) | | | Progression group (n = 33) | | | *p* value |
|  | Median | IQR | Range | Median | IQR | Range |  |
| TLG | 19.37 | 11.40-97.55 | 4.50-291.60 | 50.20 | 28.80-121.15 | 7.50-2299.04 | 0.047 |
| GLSZM_GLNU | 3.95 | 3.26–9.81 | 1.22–25.61 | 8.40 | 4.35–16.99 | 2.57–58.98 | 0.012 |
| GLRLM_RLNU | 181.14 | 108.17-385.59 | 41.24-1245.64 | 320.37 | 191.15-674.10 | 62.26-9349.86 | 0.043 |
| *IQR*, interquartile range; TLG, total lesion glycolysis; *GLSZM*, grey-level size-zone matrix; *GLNU*, grey-level non-uniformity; *GLRLM*, grey-level run-length matrix; *RLNU*, run-length non-uniformity | | | | | | | |

| **Supplemental Table 3.** Comparison of SUV-related parameters including SUVmax and SUVmean of liver between pre- and post-Combat harmonization | | | | | | | | | | | | | | |
| --- | --- | --- | --- | --- | --- | --- | --- | --- | --- | --- | --- | --- | --- | --- |
| Parameter | Pre-Combat harmonization | | | | | | | Post-Combat harmonization | | | | | | |
|  | Discover 600M scanner (n=27) | | | Discover MI scanner (n=25) | | | *p* value | Discover 600M scanner (n=27) | | | Discover MI scanner (n=25) | | | *p* value |
|  | Median | IQR | Range | Median | IQR | Range |  | Median | IQR | Range | Median | IQR | Range |  |
| SUVmax | 3.64 | 3.44-3.97 | 2.29-4.71 | 2.68 | 2.52-2.85 | 1.94-3.79 | <0.001 | 3.19 | 3.02-3.48 | 2.02-4.12 | 3.17 | 2.98-3.38 | 2.29-4.50 | 0.58 |
| SUVmean | 2.61 | 2.50-2.78 | 1.74-3.47 | 2.24 | 2.11-2.37 | 1.71-3.07 | <0.001 | 2.41 | 2.32-2.56 | 1.65-3.16 | 2.46 | 2.31-2.61 | 1.84-3.42 | 0.78 |
| *IQR*, interquartile range | | | | | | | | | | | | | | |


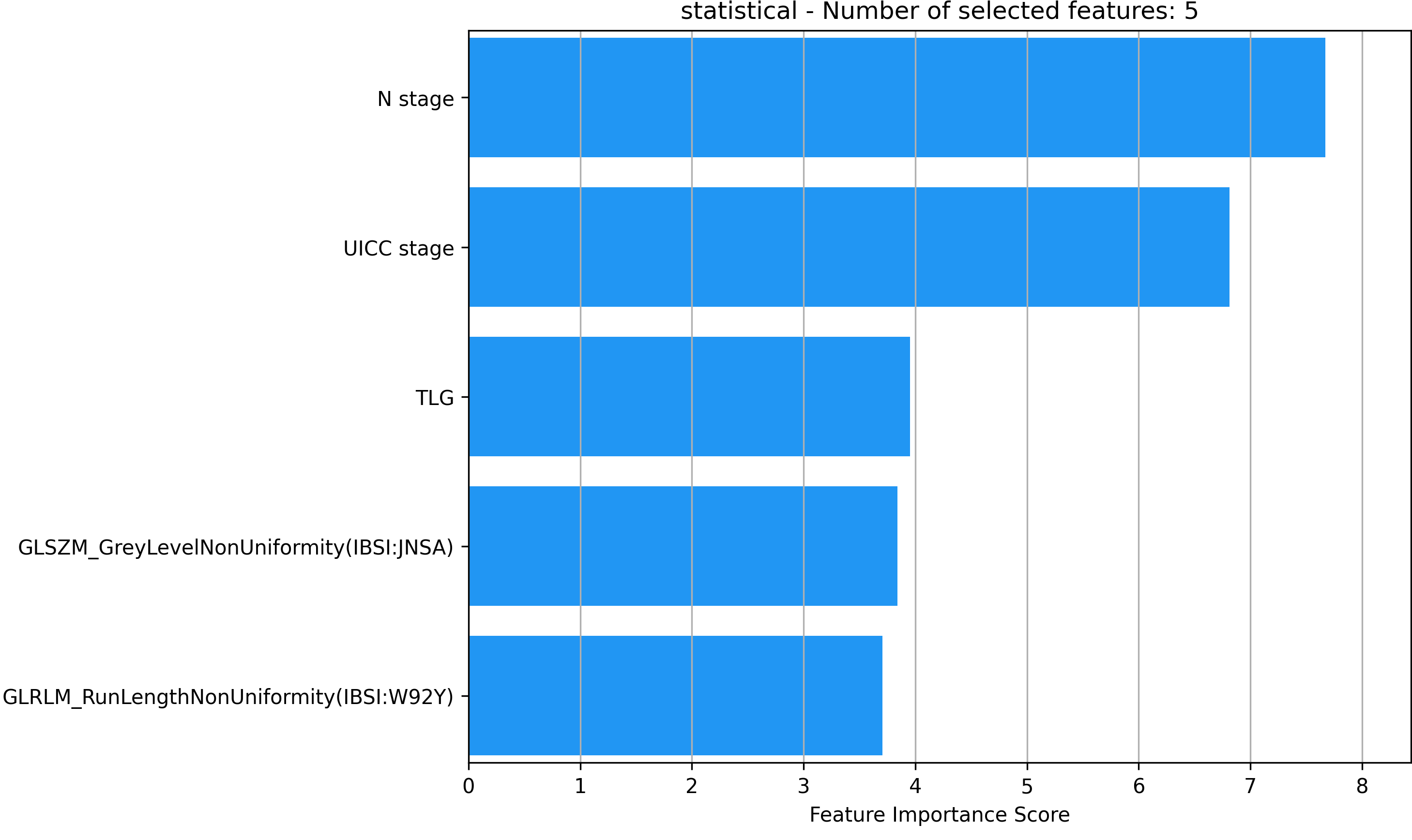


**Supplementary Figure 1** The features identified based on their importance scores using the statistical feature selection method.

N stage, UICC stage, TLG, GLSZM_GLNU and GLRLM_RLNU were selected as the important features for developing ML models.


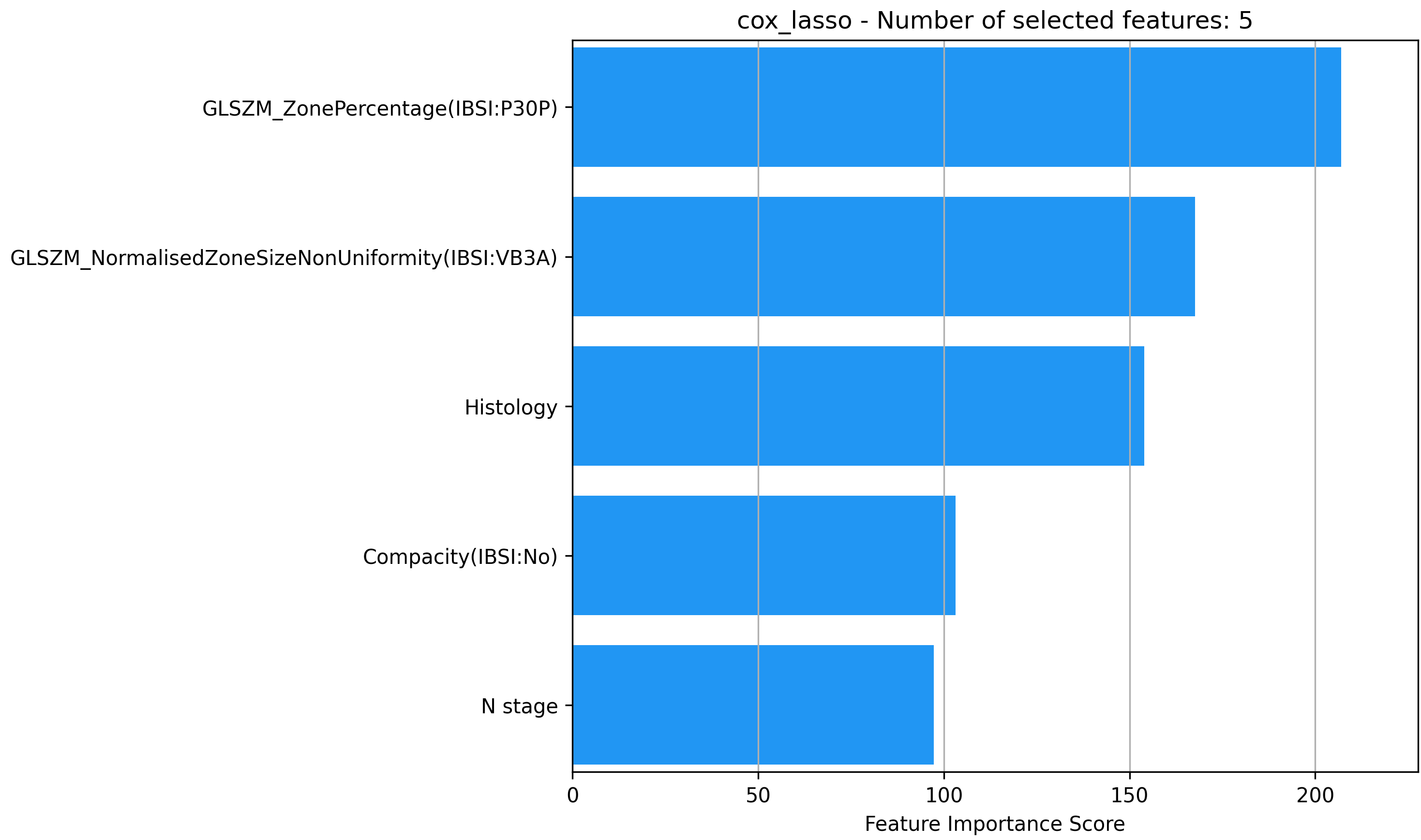


**Supplementary Figure 2** The features identified based on their importance scores using the LASSO Cox regression method.

GLSZM_zone percentage, GLSZM_normalized zone-size nonuniformity, Histology, Compacity and N stage were selected as the important features for developing ML models.

**Supplementary Figure 3** The features identified based on their importance scores using the RFE method.


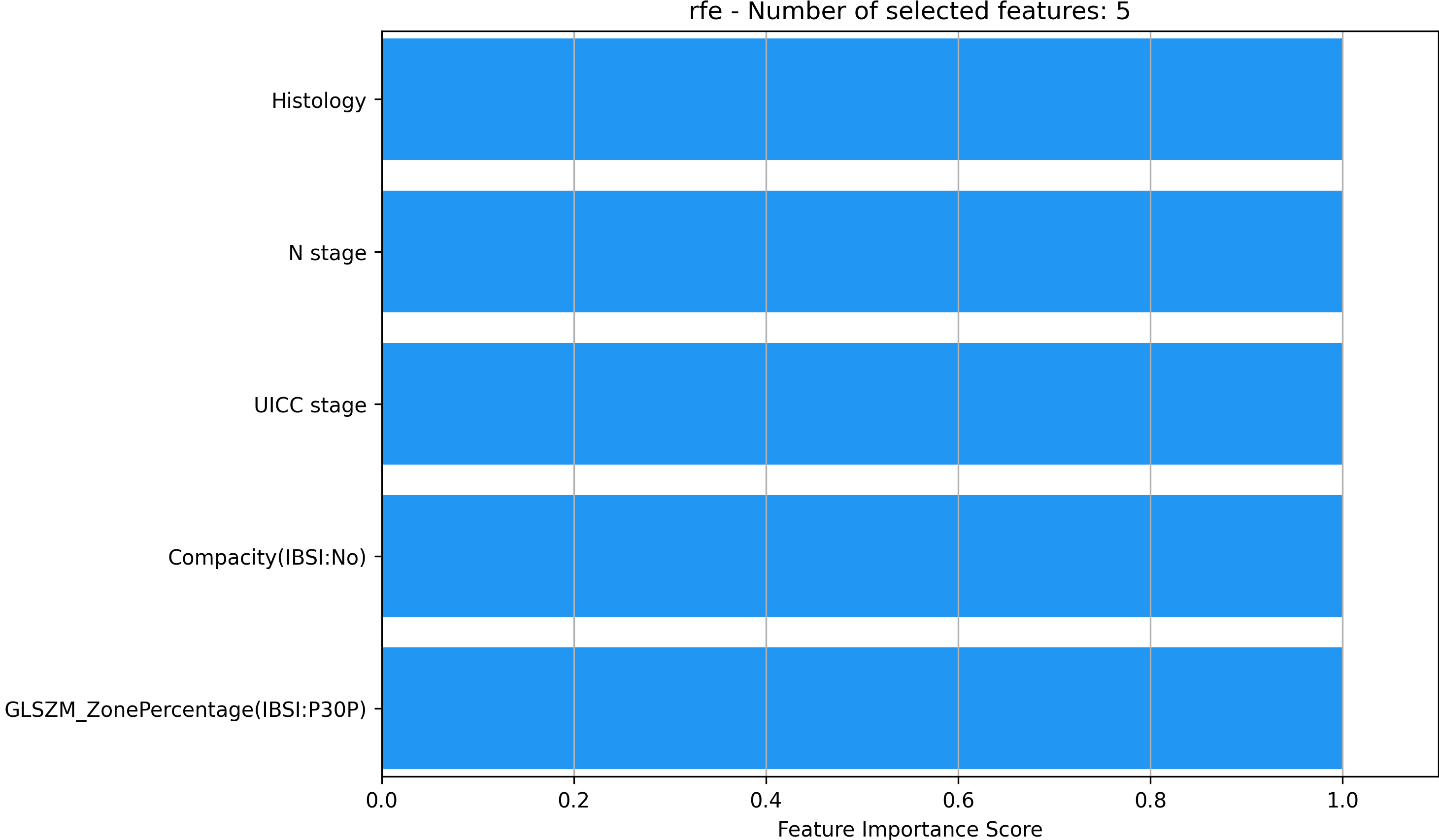


Histology, N stage, UICC stage, Compacity and GLSZM_zone percentage were selected as the important features for developing ML models.

(a)


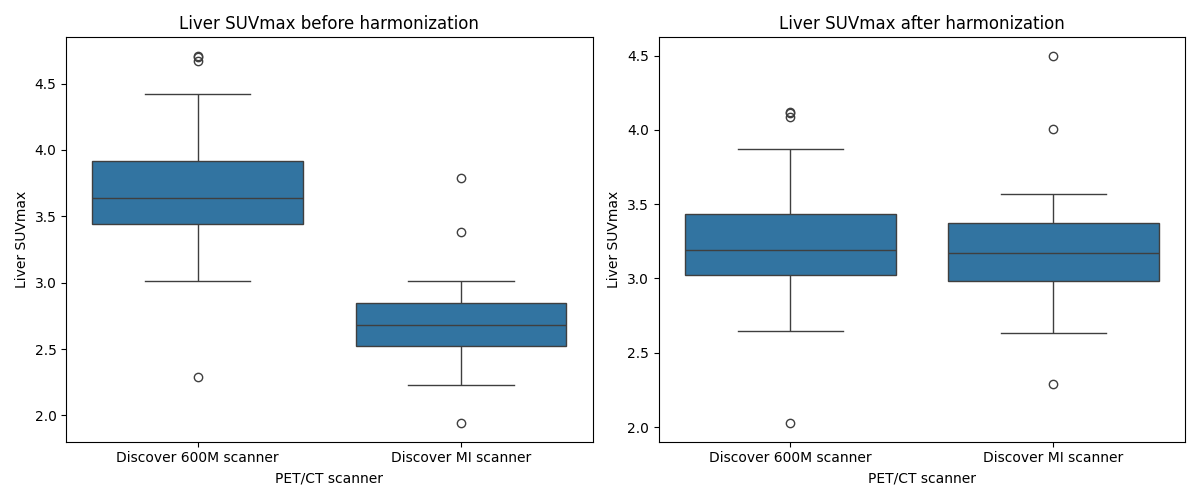


(b)


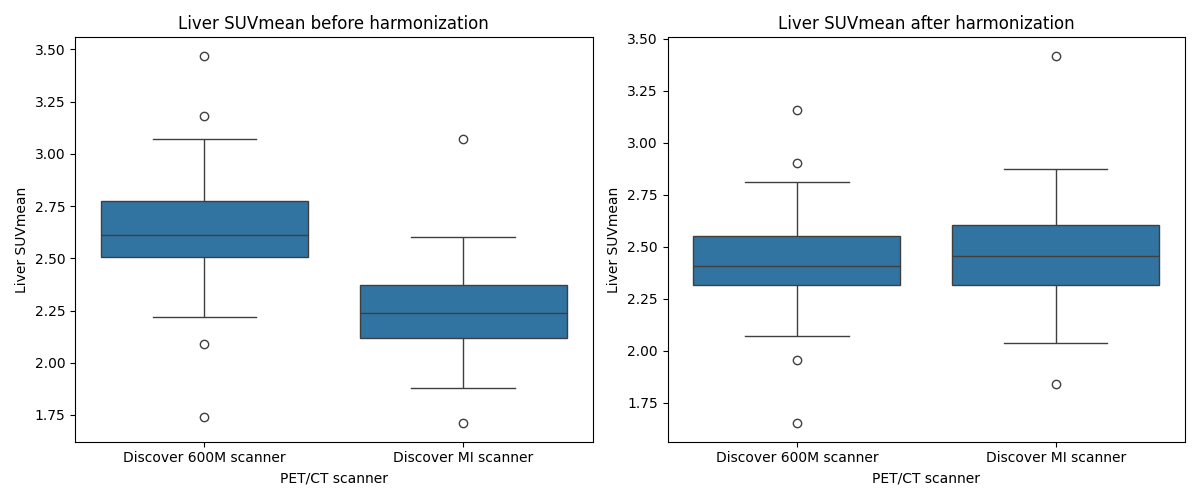


**Supplemental Figure 4**

Box plots of liver SUVmax (a) and liver SUVmean (b) between 2 PET/CT scanners pre- and post-Combat harmonization.

(a)


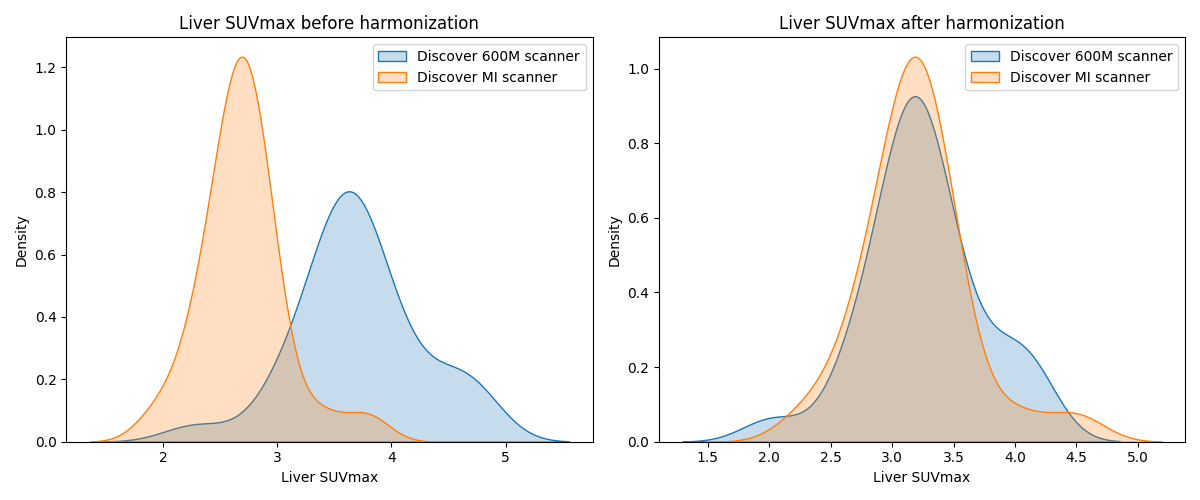


(b)


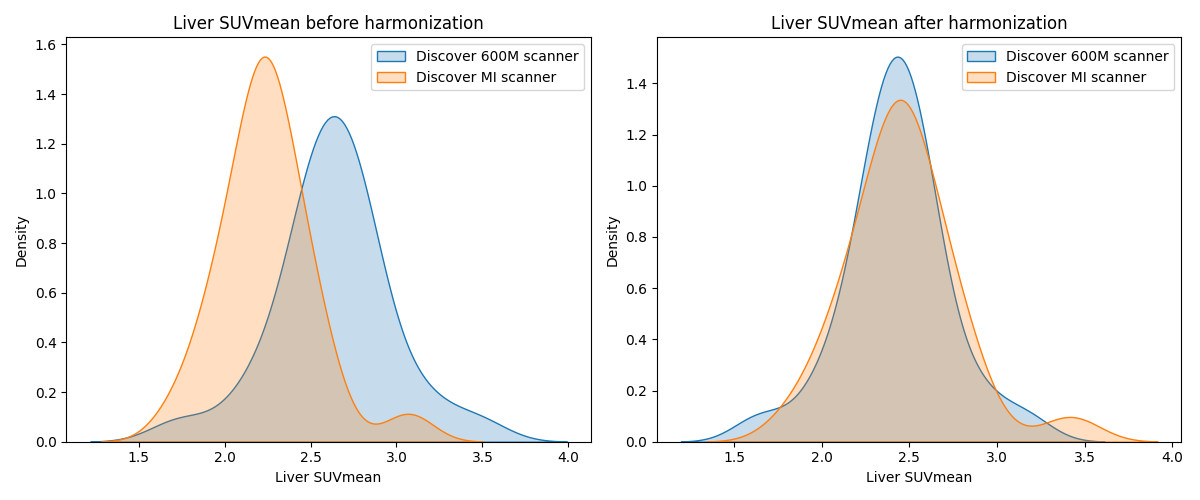


**Supplemental Figure 5**

Probability density function (%) of liver SUVmax (a) and liver SUVmean (b) pre- and post-Combat harmonization.

**Supplemental Material**

**Treatment**

All patients underwent surgery or received chemotherapy. For early stage (T1 and T2 stage) gallbladder cancer, simple cholecystectomy or radical surgery involving gallbladder removal with en bloc resection of the adjacent liver parenchyma with peri-portal and retroduodenal lymphadenectomy was considered. For locally advanced gallbladder cancers with resectable tumors, either radical surgery or neoadjuvant chemotherapy, followed by radical surgery, was considered. Systemic chemotherapy was considered for unresectable gallbladder cancers [1, 2].

All chemotherapy regimens, including neoadjuvant, adjuvant therapy, and systemic chemotherapy, involved gemcitabine alone, TS-1 alone, or combined gemcitabine plus cisplatin, gemcitabine plus TS-1 [1, 2]. For patients who received neoadjuvant chemotherapy, surgical procedures were performed within 2 months after the completion of neoadjuvant chemotherapy. Patients at a high risk of locoregional recurrence, which was associated with pT3-4 tumors, positive resection margins, or positive metastatic lymph nodes, received postoperative adjuvant chemotherapy [1].

Among the 52 eligible patients, 6, 10, 6, 14, 2, and 14 patients presented with stage I, II, IIIA, IIIB, IVA, and IVB disease, respectively. After [^18^F]-FDG-PET/CT scan, the patients underwent surgery and/or received adjuvant chemotherapy, or chemotherapy alone within 1–77 (mean: 26) days. Thirty-five patients received surgical treatment. Among them, one received preoperative neoadjuvant chemotherapy, and 14 patients subsequently received postoperative adjuvant chemotherapy. The 20 remaining patients did not receive adjuvant therapy. Meanwhile, 17 (33%) patients received chemotherapy alone.

**Default settings of each ML algorithm**

For the CPH model, we used the default settings without any specific hyperparameter tuning. The RSF algorithm is an extension of the random forest model, which considers censoring [3]. For the RSF, a hyperparameter search was conducted to optimize key parameters, including the number of trees (400-2000), maximum depth (3-10), and the minimum number of samples required at a leaf node (5-20). This search ensured that the model was tuned for optimal performance. The development of the ML model was performed on the training cohort, and the testing cohort was reserved for estimating the final predictive performances.

**ComBat harmonization methods**

The ComBat model [4] assumes that the value of each feature y measured in VOI j and scanner i can be written as:

$y_{ij}$ =α + $X_{ij}$β + $\gamma_{i}$ + $\delta_{i}$+ $\varepsilon_{\mathrm{ij}}$

α corresponds to the average value of the feature of interest *y*, *X* is the design matrix for the covariates of interest, β is the vector of regression coefficients corresponding to each covariate, $\gamma_{i}$ is the additive effect of scanner *i* affecting the measurement, $\delta_{i}$ is the multiplicative scanner effect, and $\varepsilon_{\mathrm{ij}}$is an error term [5].

ComBat harmonization consists in estimating $\gamma_{i}$ and $\delta_{i}$ using posterior means of empirical Bayes formulation (noted $\gamma_{i}^{*}$and$\delta_{i}^{*}$). The normalized value of feature *y* for VOI j and scanner *i* is then obtained as:

$y_{ij}^{Combat}=\frac{y_{ij}-\hat{\alpha}-X_{ij}\hat{\beta}-\gamma_{i}^{*}}{\delta_{i}^{*}}$+ $\hat{\alpha}$ + $X_{ij}\hat{\beta}$

where $\hat{\alpha}$and $\hat{\beta}$ are estimators of parameters α and β, respectively.

The harmonization determines a transformation for each feature separately based on the batch (here, scanner) effect observed on feature values. We used ComBat without accounting for any biological covariate (i.e., *X* = 0) because there were no differences between scanners in terms of clinical parameters (Supplemental table 4).

**Setting of VOI for measurement of SUV-related parameters of liver**

The third radiologist located a spheric VOI of about 25cm^3^ in each patient of right posterior lobe of the liver [6].

**References**

1. Aloia TA, Járufe N, Javle M, et al. Gallbladder cancer: expert consensus statement. HPB (Oxford). 2015;17:681-90.
2. Zaidi MY, Maithel SK. Updates on gallbladder cancer management. Curr Oncol Rep. 2018;20:21.
3. Ishwaran H, Gerds TA, Kogalur UB, Moore RD, Gange SJ, Lau BM. Random survival forests for competing risks. Biostatistics. 2014;15:757-73.
4. Johnson WE, Li C, Rabinovic A. Adjusting batch effects in microarray expression data using empirical Bayes methods. Biostatistics. 2007;8:118–27.
5. Fortin JP, Parker D, Tunc B, Watanabe T, Elliott MA, Ruparel K, et al. Harmonization of multi-site diffusion tensor imaging data. NeuroImage. 2017;161:149–70.
6. Orlhac F, Boughdad S, Philippe C, et al. A postreconstruction harmonization method for multicenter radiomic studies in PET. J Nucl Med. 2018:59:1321-8.
